# Clinical impact of spacer placement surgery with expanded polytetrafluoroethylene sheet for particle therapy

**DOI:** 10.1186/s13014-023-02359-5

**Published:** 2023-10-24

**Authors:** Ryosuke Fujinaka, Shohei Komatsu, Kazuki Terashima, Yusuke Demizu, Satoshi Omiya, Masahiro Kido, Hirochika Toyama, Sunao Tokumaru, Tomoaki Okimoto, Takumi Fukumoto

**Affiliations:** 1 Department of Surgery, Division of Hepato-Biliary-Pancreatic Surgery, Kobe University Graduate School of Medicine, 7-5-2 Kusunoki-cho, Chuo-ku, Kobe, 650-0017 Hyogo Japan; 2 Department of Radiology, Hyogo Ion Beam Medical Center, 1-2-1 Kouto, Shingu-cho, Tatsuno, 679-5165 Hyogo Japan; 3 Department of Radiation Oncology, Hyogo Ion Beam Medical Center Kobe Proton Center, 1-6-8 Minatojimaminami-machi, Chuo-ku, Kobe, 650-0047 Hyogo Japan

**Keywords:** Spacer placement surgery, Expanded polytetrafluoroethylene, Particle therapy, Spacer-related complication

## Abstract

**Background:**

Spacer placement surgery is useful in particle therapy (PT) for patients with abdominopelvic malignant tumors located adjacent to the gastrointestinal tract. This study aimed to assess the safety, efficacy, and long-term outcomes of spacer placement surgery using an expanded polytetrafluoroethylene (ePTFE) spacer.

**Methods:**

This study included 131 patients who underwent ePTFE spacer placement surgery and subsequent PT between September 2006 and June 2019. The overall survival (OS) and local control (LC) rates were calculated using Kaplan-Meier method. Spacer-related complications were classified according to the National Cancer Institute Common Terminology Criteria for Adverse Events (version 5.0).

**Results:**

The median follow-up period after spacer placement surgery was 36.8 months. The 3-year estimated OS and LC rates were 60.5% and 76.5%, respectively. A total of 130 patients (99.2%) were able to complete PT. Spacer-related complications of ≥ grade 3 were observed in four patients (3.1%) in the acute phase and 13 patients (9.9%) in the late phase. Ten patients (7.6%) required removal of the ePTFE spacer.

**Conclusions:**

Spacer placement surgery using an ePTFE spacer for abdominopelvic malignant tumors is technically feasible and acceptable for subsequent PT. However, severe spacer-related late complications were observed in some patients. Since long-term placement of a non-absorbable ePTFE spacer is associated with risks for morbidity and infection, careful long-term follow-up and prompt therapeutic intervention are essential when complications associated with the ePTFE spacer occur.

**Trial registration:**

retrospectively registered.

**Supplementary Information:**

The online version contains supplementary material available at 10.1186/s13014-023-02359-5.

## Background

Particle therapy (PT), such as proton and carbon-ion therapy, is currently the most advanced form of radiotherapy and has an inherent advantage over photon radiotherapy. Unlike photon beams which decrease exponentially as a function of depth, particle beams deposit relatively minimal energy along their travel path, except for concentrated maximum energy at the end of their tissue range, known as the Bragg beak [1, 2]. These unique physical properties of PT allow the delivery of higher doses of radiation to the tumor while sparing the surrounding normal tissues, thereby potentially improving tumor eradication. Recent studies have demonstrated the clinical efficacy of PT in various types of malignant tumors [3–6].

Although the reported toxicities of PT and photon radiotherapy are comparable [7, 8], the sharper dose distribution of PT needs to be carefully handled considering uncertainties and tissue changes, which potentially exposes the organs at risk such as the gastrointestinal tract to doses that exceed their constraints. This may result in significant side effects such as intestinal adhesions, gastrointestinal bleeding, or intestinal perforations when escalating the dose to the target [9]. As gastroenteric toxicity following PT can represent a negative prognostic factor, radiation dose reduction may be required, potentially leading to local recurrence [10].

To avoid these problems, space-making strategies, such as space-making particle therapy (SMPT), have been suggested, wherein medical materials are used to separate the tumor from the adjacent gastrointestinal tract to safely increase the delivered dose to the target tumor. Our previous research has demonstrated the preliminary effectiveness and prospects of SMPT in treating various types of abdominopelvic malignant tumors [11–17]. We employed an expanded polytetrafluoroethylene (ePTFE) sheet as a surgical spacer. In recent years, the application of various spacers in PT has been progressing, such as gel spacers for prostate cancer, silicon spacers for sacral chordoma, and biologic mesh spacers for pelvic tumors [18–21]. However, these studies have been limited in sample size and the long-term outcomes of spacer placement surgery have not been reported.

This study aimed to evaluate the feasibility of spacer placement surgery using ePTFE sheets in terms of procedure validation and patient tolerance. Furthermore, this study investigated the safety, efficacy, and long-term outcomes of SMPT with an ePTFE spacer in advanced abdominopelvic malignant tumors.

## Methods

### Patient characteristics

This retrospective study was conducted between September 2006 and June 2019. A total of 131 patients underwent SMPT comprising surgical spacer placement at Kobe University Hospital and Kobe Kaisei Hospital and subsequent PT at Hyogo Ion Beam Medical Center. The ePTFE sheets were used as surgical spacers in all patients. Patients who fulfilled the inclusion criteria and provided written informed consent for surgical spacer placement and subsequent PT were enrolled. The inclusion criteria for this study were as follows: (1) abdominopelvic malignant tumor confirmed histologically or clinically by diagnostic imaging, such as computed tomography (CT) and/or magnetic resonance imaging (MRI); (2) malignant tumor not suitable for upfront surgery or considered difficult to control with other local therapies; (3) malignant tumor located broadly adjacent to the gastrointestinal tract and determined to be unsuitable for delivering an adequate curative dose of PT; (4) an Eastern Cooperative Oncology Group Performance Status score of 0–2; (5) adequate organ function; and (6) no active concomitant malignancy. The treatment strategy was to place a surgical spacer in the first stage of the operation to shield the gastrointestinal tract from the irradiation field and allow subsequent PT with curative doses in the second stage.

This study was conducted in accordance with the ethical standards of the Declaration of Helsinki and was approved by the ethics committees of Kobe University Hospital (approval number: B220058), Kobe Kaisei Hospital, and Hyogo Ion Beam Medical Center.

### Spacer placement surgery

For the surgical procedure, all patients underwent general anesthesia with tracheal intubation and open laparotomy. A midline incision was made, with direct access to the abdominal cavity. After laparotomy, no distant metastasis was confirmed prior to spacer placement surgery. Spacer placement was performed to maintain an adequate distance of at least 10 mm between the tumor and the gastrointestinal tract in all directions to ensure a safe subsequent PT. For each patient, the ePTFE spacer was trimmed according to the intraoperative findings from a 20 × 15-cm ePTFE sheet with a width of 2 mm, by folding the sheet and shaping it with scissors. An appropriate number of ePTFE sheets was superimposed and sutured to fit the expected space. This method allows the surgeon to determine the final desired width of the spacer based on the patient’s anatomy. The modified ePTFE spacer was placed between the tumor and adjacent gastrointestinal tract and fixed tightly to the surrounding tissue to avoid migration [11–17]. An additional procedure, such as omental implantation and proctostomy, was added as appropriate in case of insufficient spacer volume or difficulty in covering the entire tumor with the ePTFE spacer. Intestinal resection was performed in cases of intraoperative small intestine injury, severe adhesions, or tumor invasion between the tumor and small intestine. In intraoperative cases when the colon had to be resected, spacer placement surgery was deemed not indicated to avoid infection. The relative positions of the spacer, gastrointestinal tract, and tumor were confirmed by intraoperative CT examination. No part of the tumor was resected because surgical spacer placement was a preliminary step for PT. After the postoperative course, patients were referred to the Hyogo Ion Beam Medical Center for subsequent PT.

### Treatment protocol of particle therapy

Subsequent PT was planned using a CT-based 3-dimensional treatment planning system (Xio-M system; CMS, St Louis, MO, USA; Mitsubishi Electric Corporation, Tokyo, Japan). The gross tumor volume (GTV), defined as the target primary tumor, and organs at risk were delineated on fusion images with contrast-enhanced CT and MRI. The clinical target volume (CTV) consisted of the GTV with a uniform 5-mm basic margin, while the planning target volume (PTV) was defined as the CTV plus a setup margin (5 mm). An internal margin (1 mm) was also added under the respiratory gating system [22]. The dose intensity and risk for radiation-induced adverse events were assessed using dose-volume histograms in all patients. The beam setting was chosen to facilitate the maximum possible irradiation to the GTV, CTV, and PTV, while maintaining the maximum dose applied to every 0.5 cc of the gastrointestinal tract under 48 Gy (relative biological effectiveness [RBE]) and the maximum dose to the spinal code under 45 Gy (RBE) at the Hyogo Ion Beam Medical Center [4, 23, 24].

The patients were treated using 150-MeV or 210-MeV proton beams or 320-MeV carbon-ion beams. The selection between proton therapy or carbon-ion therapy was based on the dose distribution. We used the same dose constraints for organs at risk for both proton and carbon-ion therapy. Therefore, the beam type that achieved better target coverage was selected for each patient after a discussion among several radiation oncologists [10, 25, 26]. The RBE values for protons and carbon ions at the Hyogo Ion Beam Medical Center are 1.1 and 2–3.7, respectively, depending on the depth of the spread-out Bragg peak [27].

**Fig. 1:**
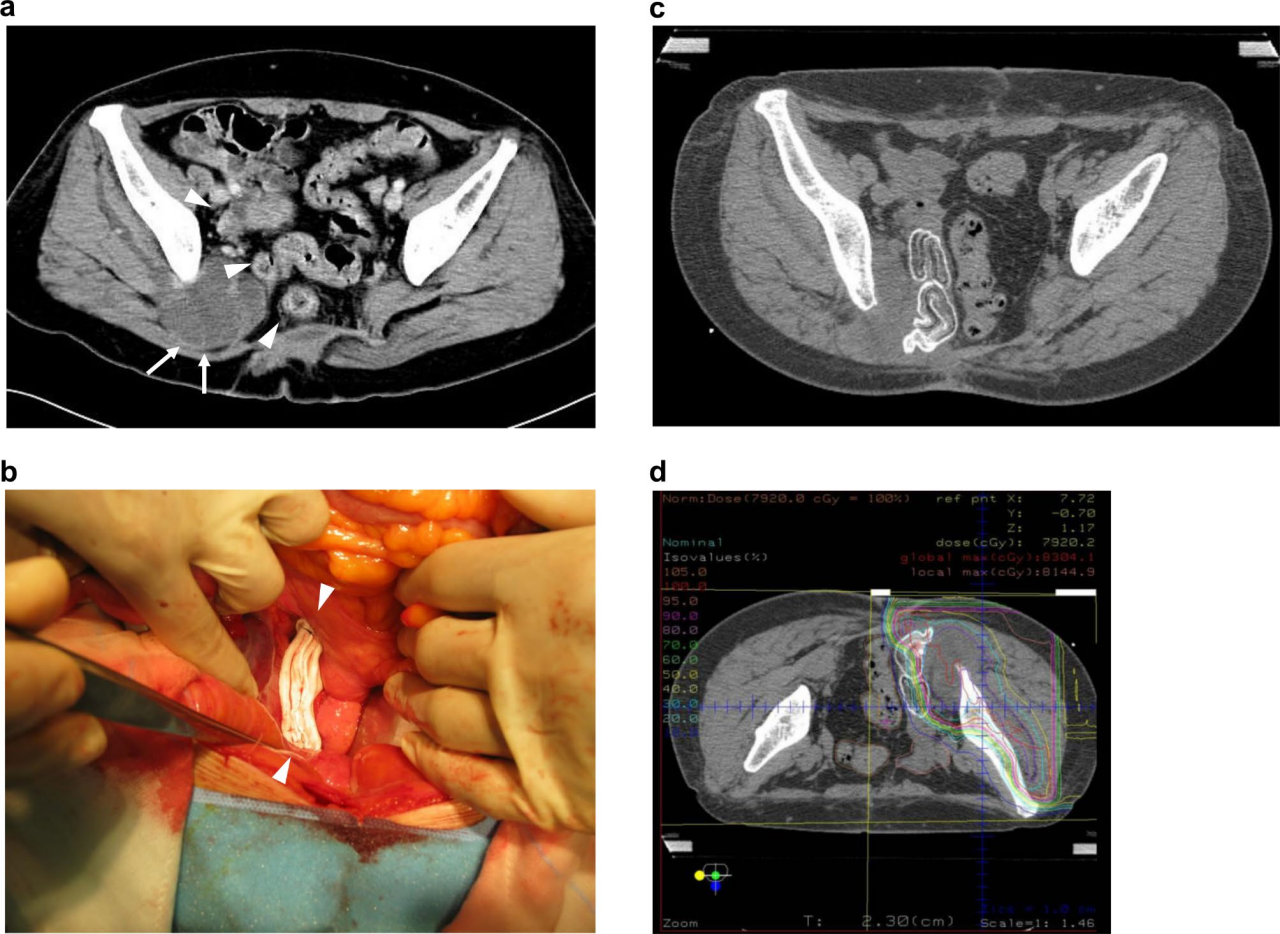
Images of a representative patient who underwent expanded polytetrafluoroethylene (ePTFE) spacer placement surgery. **a** Recurrence of chondrosarcoma (white arrow) broadly abuts the small intestine, sigmoid colon, and rectum (arrowhead). **b** Operative findings show that the ePTFE spacer (arrowhead) was inserted and sutured to the surrounding tissue at the left side of the tumor. **c** Postoperative computed tomography reveals that sufficient space between the tumor and the gastrointestinal tract was maintained by the ePTFE spacer. **d** The treatment plan shows that the clinical target volume was totally irradiated at more than 90% of the prescribed dose, and gastrointestinal tract was barely irradiated

Figure 1 shows a representative treatment course for SMPT. The tumor was diagnosed as a recurrent chondrosarcoma. Preoperative abdominal CT (Fig. 1a) demonstrated that the tumor (arrow) was located adjacent to the small intestine, sigmoid colon, and rectum (arrowhead). The ePTFE spacer (arrowhead) was placed between the tumor and adjacent gastrointestinal tract and fixed tightly to the surrounding tissue (Fig. 1b). Abdominal CT following spacer placement surgery revealed that sufficient space was maintained by the ePTFE spacer between the tumor and the gastrointestinal tract (Fig. 1c). The gastrointestinal tract was removed from the irradiation field, and the CTV was irradiated at > 90% of the prescribed dose (Fig. 1d).

### Follow-up and evaluation criteria

Patients were observed at 3–4 months for 3 years after subsequent PT and every 6 months thereafter. Follow-up was continued until patient’s death. Regular follow-up studies included physical examinations, diagnostic imaging (CT and/or MRI), and blood tests. Local recurrence was defined as radiographic enlargement of the primary tumor or appearance of new tumors inside the irradiation field. The National Cancer Institute Common Terminology Criteria for Adverse Events (version 5.0) were used to define and grade the complications of spacer placement surgery. Complications were divided into two phases: (1) the acute phase, from spacer placement surgery to the completion of subsequent PT and (2) the late phase, after PT.

### Statistical analysis

The follow-up period was calculated from the day of the spacer placement surgery. Continuous variables were reported as median and range. Categorical variables were reported as count and percentage. The overall survival (OS) and local control (LC) rates were estimated using Kaplan-Meier method. All data were analyzed using JMP 16 statistical package (SAS Institute, Cary, NC, USA).

## Results

### Baseline characteristics and operative details

The baseline characteristics of the patients are shown in Table 1. A total of 131 patients underwent ePTFE spacer placement and subsequent PT, including 63 men (48.1%) and 68 women (51.9%). The median age was 60 years (range: 18–82 years). Patients with various abdominopelvic tumors were enrolled in this study. The most frequent tumor type was sarcoma (31/131; 23.7%), followed by chordoma (28/131; 21.4%).

Table 2 reports the operative details of ePTFE spacer placement surgery. The median operative time was 173 (range: 78–522) minutes. The median blood loss was 70 (range: 0–1002) mL, and three patients (2.3%) required blood transfusion. The median number of ePTFE sheets used for the spacer was 2 (range: 1–17). Additional procedures were performed as follows: intestinal resection in three (2.3%), proctostomy in two (1.5%), intestinal resection with proctostomy in two (1.5%), and omental implantation in three patients (2.3%). The median postoperative hospital stay was 9 (range: 5–64) days, and the interval between the operation and the initiation of PT was 22 (range: 15–88) days. A total of 130 patients (99.2%) were able to complete the subsequent PT, except for a gynecological cancer patient whose tumor had progressed during the PT period.

**Table 1:** Characteristics of patients treated with ePTFE spacer placement surgery for SMPT

	No. of patients (n = 131)
Age, years, *median (range)*	60 (18–82)
Gender, *n* (%)	
Male	63 (48.1)
Female	68 (51.9)
Objects of SMPT, *n* (%)	
Hepatocellular carcinoma	10 (7.6)
Hilar cholangiocarcinoma	6 (4.6)
Pancreas cancer	11 (8.4)
Metastatic liver tumor	2 (1.5)
Local recurrence of rectal cancer	21 (16.0)
Chordoma	28 (21.4)
Chondrosarcoma	7 (5.3)
Sarcoma	31 (23.7)
Rectal gastrointestinal stromal tumor	1 (0.8)
Gynecological cancer	9 (6.9)
Urological cancer	2 (1.5)
Others	3 (2.3)
Dose fraction, *n* (%)	
50.0 GyE/25 fr	3 (2.3)
64.0 GyE/8 fr	7 (5.3)
64.0 GyE/16 fr	2 (1.5)
67.5 GyE/25 fr	6 (4.6)
70.2 GyE/26 fr	6 (4.6)
70.4 GyE/16 fr	30 (22.9)
70.4 GyE/32 fr	35 (26.7)
72.6 GyE/22 fr	2 (1.5)
74.0 GyE/37 fr	12 (9.2)
76.0 GyE/20 fr	9 (6.9)
76.0 GyE/38 fr	2 (1.5)
80.0 GyE/20 fr	3 (2.3)
Others	13 (9.9)
Discontinuation	1 (0.8)
Beam type, *n* (%)	
Carbon ion	46 (35.1)
Proton	85 (64.9)

*ePTFE* expanded polytetrafluoroethylene, *SMPT* space-making particle therapy, *GyE* gray equivalent, *fr* fraction

**Table 2:** Operative details of ePTFE spacer placement surgery

	No. of patients (n = 131)
**Operative outcomes**	
Operative time, min, *median (range)*	173 (78–522)
Blood loss, ml, *median (range)*	70 (0–1002)
Need for blood transfusion, *n* (%)	3 (2.3)
Number of ePTFE sheets, sheets, *median (range)*	2 (1–17)
Additional procedure, *n* (%)	
Intestinal resection	3 (2.3)
Proctostomy	2 (1.5)
Intestinal resection with proctostomy	2 (1.5)
Omental implantation	3 (2.3)
**Perioperative outcomes**	
Postoperative hospital length of stay, days, *median (range)*	9 (5–64)
Time between operation and initiation of particle therapy, days, *median (range)*	22 (15–88)
Completion of curative subsequent PT, *n* (%)	130 (99.2)

*ePTFE* expanded polytetrafluoroethypene, *PT* particle therapy

### Local control and survival outcomes with space-making particle therapy

**Fig. 2:**
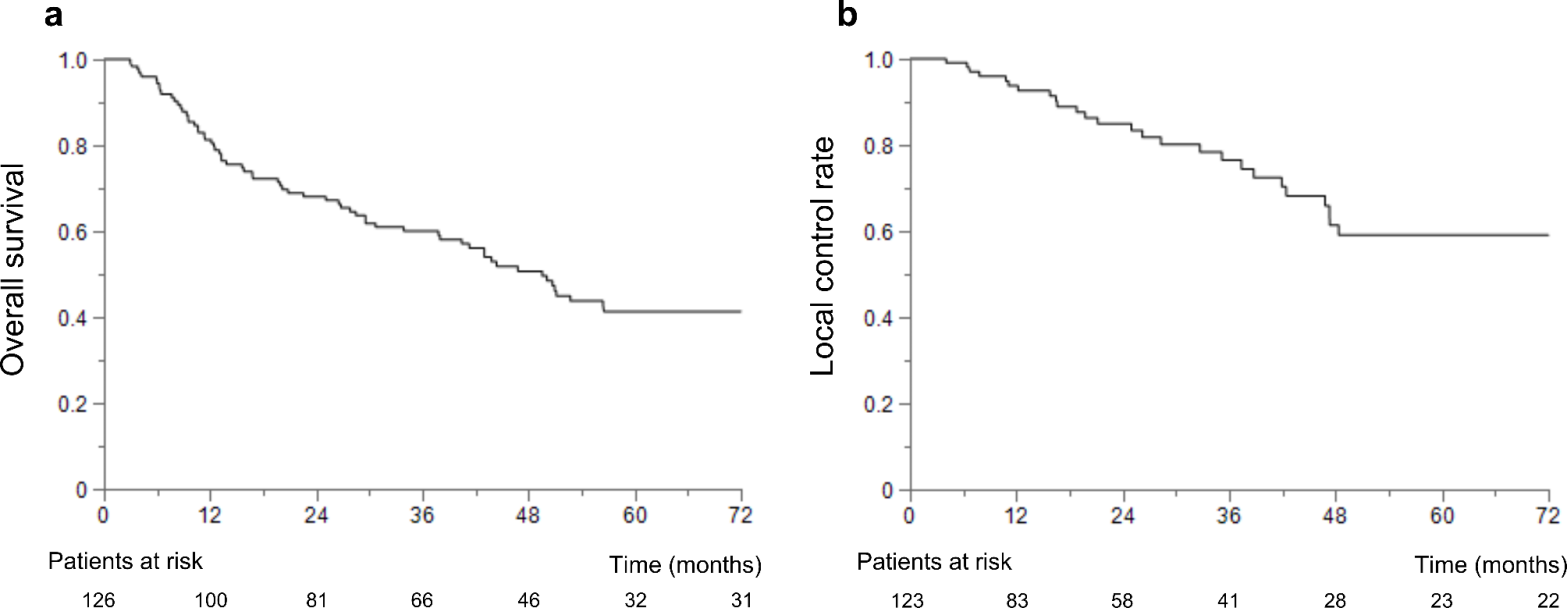
Survival curves of patients who underwent expanded polytetrafluoroethylene spacer placement surgery for space-making particle therapy. **a** Overall survival and **b** local control rate

The median follow-up period was 36.8 (range: 3.0–163.6) months. The OS and LC rates are shown in Fig. 2. The median survival time was 49.5 months, and the 3- and 5- year OS rates were 60.5% and 41.3%, respectively. The 3- and 5-year LC rates were 76.5% and 59.1%, respectively.

A total of 27 patients (20.6%) presented with local recurrences. The median interval from spacer placement surgery to local recurrence was 25.0 (range: 4.1–126.1) months.

### Complication related to spacer placement surgery

Complications related to ePTFE spacer placement surgery are shown in Table 3. Eight intraoperative complications were observed in seven patients (5.3%). Four patients (3.1%) showed insufficient spacer placement, and four patients (3.1%) had damage to the small intestine during spacer placement surgery. Postoperative complications were observed in 34 patients (26.0%) in the acute phase and 15 patients (11.5%) in the late phase, and 10 patients (7.6%) required removal of the ePTFE spacer. In the acute phase, four patients (3.1%) had complications of ≥ grade 3. Among the four patients, one had ileus, one gastrointestinal perforation, one intestinal necrosis, and one internal hernia. The patient with ileus was treated conservatively using a long intestinal tube. The other three patients required reoperation, and removal of the ePTFE spacer was needed in two patients who had gastrointestinal perforation and intestinal necrosis. Two patients requiring removal of the ePTFE spacer could receive subsequent PT because these two patients underwent omental implantation and/or proctostomy simultaneously with spacer removal.

**Table 3:** Complications of ePTFE spacer placement surgery

	No. of patients (n = 131)
**Intraoperative complications**, ***n (%)***	
None	124 (94.7)
Insufficient placement of ePTFE spacer	4 (3.1)
Damage to small intestine	4 (3.1)
**Acute phase complications**^a^, ***n (%)***	
None	97 (74.0)
Surgical site infection	
Grade 1–2	5 (3.8)
Grade 3–4	0 (0)
Ileus	
Grade 1–2	12 (9.2)
Grade 3–4	1 (0.8)
Gastrointestinal perforation	
Grade 1–2	0 (0)
Grade 3–4	1 (0.8)
Intestinal necrosis	
Grade 1–2	0 (0)
Grade 3–4	1 (0.8)
Internal hernia	
Grade 1–2	0 (0)
Grade 3–4	1 (0.8)
Ascites	
Grade 1–2	2 (1.5)
Grade 3–4	0 (0)
Abdominal pain related to ePTFE spacer	
Grade 1–2	9 (6.9)
Grade 3–4	0 (0)
Enteritis	
Grade 1–2	2 (1.5)
Grade 3–4	0 (0)
Urinary tract infection	
Grade 1–2	4 (3.1)
Grade 3–4	0 (0)
Pneumonitis	
Grade 1–2	1 (0.8)
Grade 3–4	0 (0)
Disseminated intravascular coagulation	
Grade 1–2	1 (0.8)
Grade 3–4	0 (0)
**Late phase complications**^a^, ***n (%)***	
None	116 (88.5)
Abscess formation	
Grade 1–2	1 (0.8)
Grade 3–4	3 (2.3)
Gastrointestinal perforation	
Grade 1–2	1 (0.8)
Grade 3–4	4 (3.1)
Grade 5	1 (0.8)
Refractory skin ulcer	
Grade 1–2	0 (0)
Grade 3–4	1 (0.8)
Bladder fistula	
Grade 1–2	0 (0)
Grade 3–4	1 (0.8)
Urinary retention	
Grade 1–2	0 (0)
Grade 3–4	3 (2.3)

*ePTFE* expanded polytetrafluoroethylene

^a^ Common Terminology Criteria for Adverse Events, version 5.0

In the late phase, 13 patients (9.9%) had ≥ grade 3 complications. Among the 13 patients, three had abscess formation associated with ePTFE spacer infection, five had gastrointestinal perforation, one had refractory skin ulcer in which the spacer was exposed through the skin, one had bladder fistula in which the spacer migrated to the bladder, and three had urinary retention, possibly due to the long-term placement of the ePTFE spacer. Grade-5 complications were observed in one patient who developed gastrointestinal perforation, and the interval between spacer placement surgery and the onset of perforation was 6 months. Reoperation was required in nine patients, and the ePTFE spacer was removed in eight patients. The median duration from spacer placement surgery to reoperation due to late complications was 17.8 (range: 4.5–112.1) months.

**Fig. 3:**
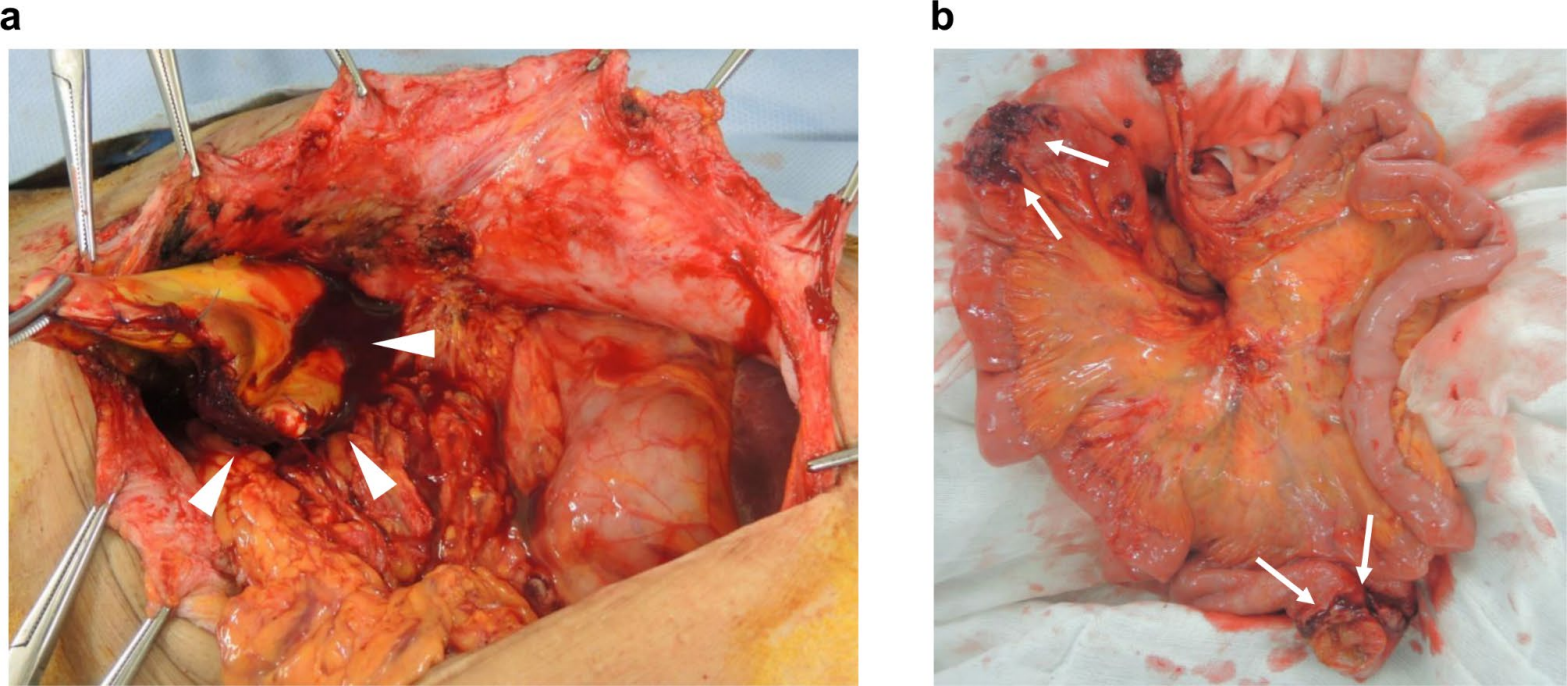
Intestinal perforation that occurred 9 years after expanded polytetrafluoroethylene (ePTFE) spacer placement surgery. **a** Operative finding shows infected ePTFE spacer after intestinal perforation (arrowhead). **b** Intestinal perforation due to long-term placement of ePTFE spacer (white arrow)

Figure 3 shows the operative findings of intestinal perforation that occurred 9 years after ePTFE spacer placement surgery. After laparotomy, the infected ePTFE spacer (arrowhead) was removed (Fig. 3a). Since the ePTFE spacer was in contact with the perforated sites of the intestines (arrow), long-term placement of foreign material was suspected as the cause of intestinal perforation (Fig. 3b).

Complications related to PT after ePTFE spacer placement surgery are shown in Supplementary Table 1, Additional file 1.

## Discussion

SMPT is a viable method designed to increase the tumor dose while limiting exposure to adjacent organs. The concept of combination treatment involving surgical spacer placement and radiotherapy has been previously applied with conventional photon radiotherapy for other malignant tumors, such as rectal cancer [28, 29]. Recent studies have demonstrated the advantages of using hydrogel rectal spacer for prostate cancer with photon radiotherapy [30, 31]. However, due to the superior physical and biological features of PT, SMPT has the following advantages over conventional combination treatments: tumors can be controlled with high probability if a sufficient PT dose is delivered to the entire tumor, and only a 10-mm distance between the tumor and adjacent organs is sufficient for the safe delivery of curative doses [11, 14]. Thus, we presume that this combined approach may have practical applications in the clinical use of PT.

This study aimed to evaluate the safety and efficacy of ePTFE spacer placement for SMPT. The reasons for selecting ePTFE sheets as surgical spacers were as follows: (1) ePTFE sheets are biocompatible materials that have been used for decades in various medical applications, such as vascular grafts, heart patches, and hernia repairs; (2) ePTFE sheets can be tailored to conform to the patient’s specific anatomy by cutting and/or superimposing the sheets; and (3) the non-absorbable material of ePTFE ensures stability in subsequent PT with no alteration in spacer volume. We observed early initiation of PT following spacer placement surgery (median interval: 22 days), high LC rate (3 years: 76.5%), and acceptable OS (median survival time: 49.5 months) in this study. We believe that ePTFE spacer placement surgery is a technically feasible and acceptable procedure, allowing for higher dose management of PT for tumors adjacent to the gastrointestinal tract and expanding the indications for PT. To the best of our knowledge, the number of cases presented in this study is the largest ever published using an ePTFE spacer for subsequent PT.

Although severe acute phase complications were observed in four patients (3.1%), subsequent PT was completed in all patients, except for one patient with tumor progression. Acute phase complications did not adversely affect subsequent PT in this study. However, severe late-phase complications occurred at a relatively high rate in 13 (9.9%) patients. While SMPT indicates the potential for long-term survival, it also highlights the risk for late-phase complications associated with prolonged placement of the ePTFE spacer, which becomes a foreign material after the completion of PT. Severe late-phase complications have been reported in the literature. Ogino et al. [32] reported the intraluminal migration of an ePTFE spacer at 2 years after spacer placement surgery. They suggested that contact between the spacer and gastrointestinal tract caused a local inflammatory reaction and promoted complete penetration of the gastrointestinal wall. It is difficult to distinguish between spacer-related and radiation-induced complications because the region near the spacer irradiated with a high dose of PT for local control and dose distribution may contain errors. However, the probability of a complication resulting from the combination of both the spacer and PT cannot be disregarded. The extended duration between the completion of PT and the occurrence of late-phase complications (median interval: 17.8 months) suggests that the long-term damage caused by mechanical irritation and the local inflammatory response triggered by the ePTFE spacer may have been the inciting factors for these complications rather than the PT. Late phase complications, such as spacer-related infection and gastrointestinal perforation, require removal of the ePTFE spacer. Shiba et al. [33] reported ePTFE spacer-related infection, resulting in colon perforation that was observed 58 months after the initiation of PT, and they underwent surgical removal of the ePTFE spacer. We removed the ePTFE spacer in eight patients who experienced severe late-phase complications. Careful long-term follow-up and prompt therapeutic intervention for complications should be emphasized, particularly for late-phase complications.

Recently, a novel bioabsorbable spacer made from polyglycolic acid (PGA) has been introduced and applied for spacer placement surgery [34]. Preclinical evaluations have reported that bioabsorbable PGA spacers have water-equivalence, biocompatibility, and thickness retention properties. It is designed to maintain 80% of their thickness for at least 8 weeks and to thereafter decrease in volume spontaneously [35]. PGA spacer is available for clinical use in combination with PT in Japan [36, 37], and this novel spacer may reduce the incidence of late phase complications such as gastrointestinal perforation, spacer-related infection, and abdominal pain consequent to surgical spacer. Since the problems with the ePTFE spacer were highlighted in this study, the bioabsorbable spacer should be used for SMPT in the future.

The present study has several limitations. First, this was a single-arm retrospective cohort study without a control group. Second, although this study confirmed favorable clinical outcomes, the evaluation of OS and LC rates was difficult because spacer placement surgery was performed in patients of various backgrounds. Third, the changes in the dose distributions for the GTV, CTV, and PTV based on the dose-volume histogram owing to spacer placement were not examined. However, our previous research reported a significant improvement in dose distributions in various types of abdominopelvic malignant tumors [12, 13, 15–17]. The findings of these studies may support the validity of the current research design and outcomes. Finally, because the clinical use of absorbable spacers is available, spacers for SMPT may be replaced mainly by PGA spacers instead of ePTFE spacers in the near future.

## Conclusions

Spacer placement surgery using an ePTFE spacer for abdominopelvic malignant tumors is technically feasible and acceptable for subsequent PT. This procedure may provide favorable clinical outcomes in tumors located adjacent to the normal organs. However, the long-term placement of an ePTFE spacer is associated with risks for morbidity and infection. Since novel PGA spacers have the potential to overcome these risks, further studies are warranted to assess the applicability and safety of PGA spacers.

### Electronic supplementary material

Below is the link to the electronic supplementary material.

Additional file 1: Supplementary Table 1: Complications related to particle therapy after ePTFE spacer placement surgery

## Data Availability

The datasets used and analyzed during the current study are available from the corresponding author on reasonable request.

## List of abbreviations

CT: computed tomography
CTV: clinical target volume
ePTFE: expanded polytetrafluoroethylene
GTV: gross tumor volume
LC: local control
MRI: magnetic resonance imaging
OS: overall survival
PGA: polyglycolic acid
PT: particle therapy
PTV: planning target volume
RBE: relative biological effectiveness
SMPT: space-making particle therapy
