# Supplementary material for: Clinical impact of spacer placement surgery with expanded polytetrafluoroethylene sheet for particle therapy
Source: Radiat Oncol. 2023 Oct 24;18:173. doi: 10.1186/s13014-023-02359-5 (PMC10594906; doi:10.1186/s13014-023-02359-5)
Supplement: Supplementary file 1 — Additional file 1: Supplementary Table 1: Complications related to particle therapy after ePTFE spacer placement surgery [file 13014_2023_2359_MOESM1_ESM.docx]

**Supplementary Table 1** Complications related to particle therapy after ePTFE spacer placement surgery

|  | No. of patients (n = 131) |
| --- | --- |
| Acute phase complications^a^, *n* (%) |  |
| None | 52 (39.7) |
| Radiation dermatitis |  |
| Grade 1–2 | 74 (56.5) |
| Grade 3–4 | 6 (4.6) |
| Gastrointestinal disturbance |  |
| Grade 1–2 | 1 (0.8) |
| Grade 3–4 | 0 (0) |
|  |  |
| Late phase complications^a^, *n* (%) |  |
| None | 91 (69.5) |
| Radiation dermatitis |  |
| Grade 1–2 | 16 (12.2) |
| Grade 3–4 | 4 (3.1) |
| Muscle inflammation |  |
| Grade 1–2 | 3 (2.3) |
| Grade 3–4 | 0 (0) |
| Neurological disorder |  |
| Grade 1–2 | 11 (8.4) |
| Grade 3–4 | 2 (1.5) |
| Bone fracture |  |
| Grade 1–2 | 4 (3.1) |
| Grade 3–4 | 0 (0) |
| Pain |  |
| Grade 1–2 | 5 (3.8) |
| Grade 3–4 | 0 (0) |
| Urinary retention |  |
| Grade 1–2 | 2 (1.5) |
| Grade 3–4 | 0 (0) |
| Lymphedema |  |
| Grade 1–2 | 2 (1.5) |
| Grade 3–4 | 1 (0.8) |
| Pleural effusion |  |
| Grade 1–2 | 1 (0.8) |
| Grade 3–4 | 0 (0) |
| Gastrointestinal disturbance |  |
| Grade 1–2 | 3 (2.3) |
| Grade 3–4 | 1 (0.8) |

*ePTFE* expanded polytetrafluoroethylene

^a^ Common Terminology Criteria for Adverse Events, version 5.0
